# Functionalized carboxylate deposition of triphenylamine-based organic dyes for efficient dye-sensitized solar cells

**DOI:** 10.1039/c8ra06595k

**Published:** 2018-09-17

**Authors:** Md Ataul Mamun, Qiquan Qiao, Brian A. Logue

**Affiliations:** Department of Chemistry and Biochemistry, South Dakota State University Brookings SD 57007 USA brian.logue@sdstate.edu +1-605-688-6698; Department of Electrical Engineering and Computer Science, South Dakota State University Brookings SD 57007 USA

## Abstract

The standard dip-coating dye-loading technique for dye-sensitized solar cells (DSSCs) remains essentially unchanged since modern DSSCs were introduced in 1991. This technique constitutes up to 80% of the DSSC fabrication time. Dip-coating of DSSC dyes not only costs time, but also generates a large amount of dye waste, necessitates use of organic solvents, requires sensitization under dark conditions, and often results in inefficient sensitization. Functionalized Carboxylate Deposition (FCD) was introduced as an alternative dye deposition technique, requiring only 2% of the fabrication time, eliminating the need for solvents, and significantly reducing dye waste. In this study, FCD was used to deposit two relatively large triphenylamine-based organic dyes (L1 and L2). These dyes were sublimated and deposited in <20 minutes *via* a customized FCD instrument using a vacuum of ∼0.1 mTorr and temperatures ≤280 °C. FCD-based DSSCs showed better efficiency (*i.e.*, 5.03% and 5.46% for L1 and L2 dyes, respectively) compared to dip-coating (*i.e.*, 4.36% and 5.35% for L1 and L2, respectively) in a fraction of the deposition time. With multiple advantages over dip-coating, FCD was shown to be a viable alternative for future ultra-low cost DSSC production.

## Abstract 2

Functionalized carboxylate deposition involves deposition of molecules from the gas phase and is an alternative dye loading technique to dip-coating. It was used to create a monolayer of large molecular weight dyes on TiO_2_, providing multiple advantages to dip-coating.

## Graphical abstract

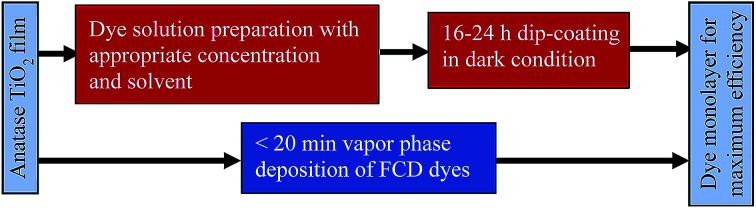

## 1. Introduction

Dye sensitized solar cells (DSSCs) are produced from low-cost materials, their fabrication does not require clean room/glove-box conditions, and DSSCs have produced power conversion efficiencies (PCEs) of more than 11%.^1–3^ Although the advantages of DSSCs are promising, their fabrication requires some high-cost materials, long fabrication times, and the use of organic solvents. For example, organometallic dyes are commonly used to produce high efficiency DSSCs.^4,5^ These dyes typically require complex synthetic and purification processes. Although progress has been made towards higher-efficiency DSSCs, the state-of-the-art dye loading technique, dip-coating, constitutes a limiting step in their fabrication. The dip-coating method for dye loading requires the longest portion of fabrication time (an average of 16 h)^6^ and has remained essentially unchanged since the modern DSSC was introduced by O'regan and Grätzel in 1991.^7^ The conventional dye-loading technique is not only inefficient, in terms of duration, but also has other drawbacks, including requiring organic solvents,^8,9^ generating significant dye waste,^6^ and sensitization under dark conditions.^10^ Moreover, dip-coating requires a high concentration of dye, which increases the probability of dye aggregation, leading to degradation of cell performance.^4,11–13^ To counteract dye aggregation in dip-coating, the dye solution is often prepared with co-adsorbents (*e.g.*, cholic acid)^14^ further complicating the fabrication process.^15^ Furthermore, after multiple uses of a single dye solution for dip-coating, the dye solution concentration changes and consistent sensitization requires cautious evaluation.

**Fig. 1:**
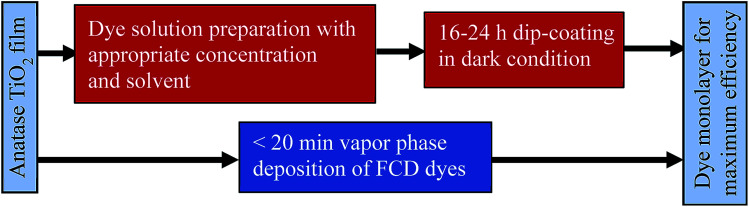
Comparison of FCD^6^ and conventional dip-coating dye loading processes.

**Fig. 2:**
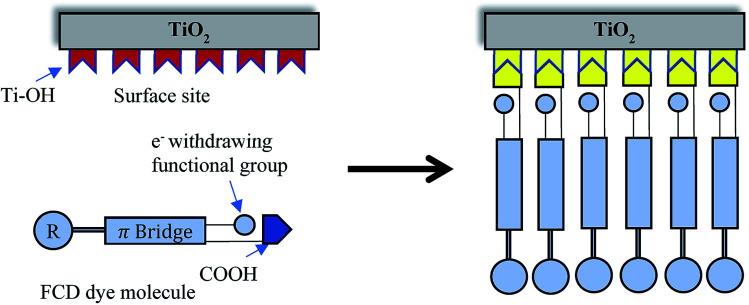
An FCD dye molecule attaches through a –COOH group with surface –OH site.

Functionalized Carboxylate Deposition (FCD), a vapor phase deposition technique, was introduced as an alternative sensitization technique in 2015.^6^ FCD significantly reduces dye loading time, allows more efficient use of dye material, and avoids the use of solvents. Fig. 1 shows the comparison of FCD with conventional dip-coating dye loading. FCD utilizes dyes containing an electron withdrawing group in the α-position to a carboxyl group. When these dyes are evaporated/sublimated, they react chemically with hydroxyl groups which dominate the surface chemistry of TiO_2_ particles.^16^ As shown in Fig. 2, the FCD dyes covalently bond to a TiO_2_ surface hydroxyl with the carboxyl group of the dye through an esterification-type reaction. For FCD sensitization, dyes need to evaporate before degrading at elevated temperatures.^6^ However, at atmospheric pressure, many organic dyes degrade before sublimating/evaporating. Thus, a moderate to high vacuum is necessary to reduce the evaporation temperature. Mallam *et al.* demonstrated vapor phase deposition of two organic dyes, (*Z*)-2-cyano-3-(4-(dimethyl-amino)phenyl)acrylic acid and (*Z*)-2-cyano-3-(4-(diphenylamino)phenyl)-acrylic acid.^6^ They achieved comparable PCEs (3.17% and 3.30%, respectively) with that of dip-coating (2.62% and 3.37%, respectively), but the two dyes used were themselves relatively inefficient.

**Fig. 3:**
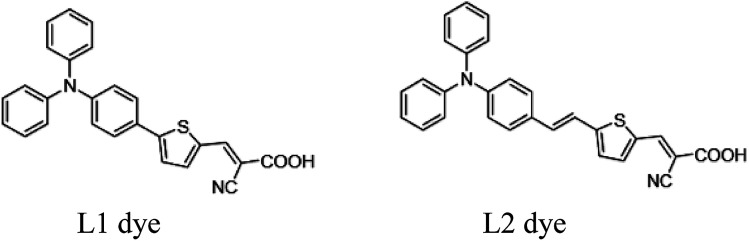
Structures of the triphenylamine-based organic dyes used in this study. L1: 5-[4-(Diphenylamino)phenyl]thiophene-2-cyanoacrylic acid and L2: 3-(5-(4-(diphenylamino)styryl)thiophen-2-yl)-2-cyanoacrylic acid.

In order for FCD to be more broadly accepted as an alternative sensitization technique to dip-coating, there is a need to demonstrate broader applicability, especially for larger dyes. Specifically, because of instrument limitations, Mallam *et al.* used a relatively high pressure of 500 mTorr to sublimate two dyes (MW < 340 g mol^−1^) at 180 and 210 °C, respectively. Here, we report the rapid (<20 min) sensitization of photoanodes with two triphenylamine-based organic dyes, L1 (5-[4-(diphenylamino)phenyl]thiophene-2-cyanoacrylic acid; MW = 422.50 g mol^−1^), and L2 (3-(5-(4-(diphenylamino)styryl)thiophen-2-yl)-2-cyanoacrylic acid; MW = 448.54 g mol^−1^) (Fig. 3), and compare the power conversion efficiencies (PCEs) of DSSCs produced *via* FCD and the conventional dip-coating method.

## 2. Experimental procedures

### 2.1. Materials

Fluorine doped tin oxide (FTO) glass substrates were purchased from Hartford Glass Co., Indiana, USA. Nanocrystalline TiO_2_ (Ti-Nanoxide T/SP), scattering TiO_2_ (Ti-Nanoxide R/SP), I_3_^−^/I^−^ electrolyte (Iodolyte HI-30), activated platinum solution (Platisol T), and thermoplastic sealant (Meltonix 1170-60) were purchased from Solaronix (Aubonne, Switzerland). Organic dyes, L1 (5-[4-(diphenylamino)phenyl]thiophene-2-cyanoacrylic acid) and L2 (3-(5-(4-(diphenylamino)styryl)thiophen-2-yl)-2-cyanoacrylic acid), were acquired from Dyenamo, Stockholm, Sweden. All materials were used as received.

### 2.2. Photoanode preparation

FTO substrates were cleaned by sonication in aqueous solutions of dodecyl sulfate sodium salt, deionized (DI) water, acetone, and 2-propanol each for 15 min, followed by UV exposure for 15 min. A layer of nanocrystalline TiO_2_ (Ti-Nanoxide T/SP, Solaronix) of 0.25 cm^2^ was deposited *via* doctor blading and then sintered at 450 °C for ∼30 min. A second nanocrystalline TiO_2_ layer was deposited according to the same procedure to achieve a thickness of ∼10 μm. A TiO_2_ light scattering layer of Ti-Nanoxide R/SP (particle size > 100 nm) was then screen printed on top of the mesoporous layer and sintered at 450 °C for 30 min. The photoanode was then treated by immersing in a 0.1 M solution of HCl for 2 h. Then the photoanode was dried with compressed N_2_ gas.

### 2.3. Functionalized carboxylate deposition

**Fig. 4:**
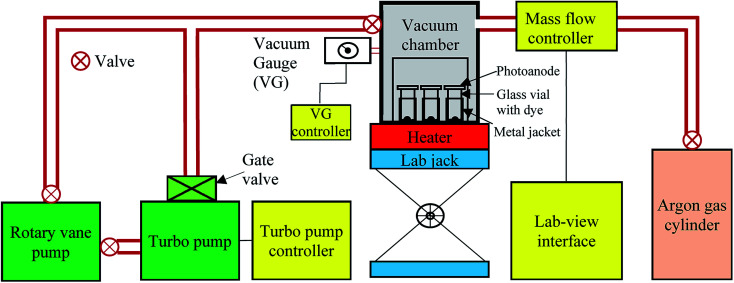
Simplified schematic diagram of the FCD instrument.

A customized FCD instrument (schematic shown in Fig. 4) consisting of a turbo pump, rotary vane pump, mass flow controller, and custom vacuum chamber was used to create 0.1 mTorr vacuum inside a 1.6 L vacuum chamber. Approximately 100 μg of FCD dye was placed into a 1.9 mL vial. The photoanode was placed on top of the glass vial with the nc-TiO_2_ in the center of the vial opening, facing into the vial. A metal jacket was placed around the vial and the vial was placed on top of an electric hot plate. With a lab-jack, the hotplate raised up to the vacuum chamber, and a vacuum was applied *via* rotary vane and turbo pumps. When a vacuum of ∼3 mTorr was reached, the hotplate was heated to ∼260 °C for the L1 dye and ∼280 °C for the L2 dye and held at that temperature for the desired amount of time. Following evaporation of the dye, the heater was turned off, and the chamber was cooled, unassisted, to 150 °C. The vacuum chamber was then filled with argon gas until atmospheric pressure was reached. The sensitized photoanodes were then rinsed sequentially with anhydrous ethanol and acetone to remove excess dye. The photoanodes were then dried with compressed N_2_ gas. Excess dye in the wash solution was recovered by allowing the ethanol and acetone to evaporate unassisted in a hood.

After the efficiency was measured, the DSSCs were deconstructed and the photoanodes were rinsed with acetonitrile to remove the residual electrolyte and then dried with compressed N_2_ gas. The dye attached with the photoanodes was extracted by dipping them in 3 mL of a 0.1 M NaOH solution for 24 h.

### 2.4. Dip-coating

For dip-coating, dye solutions (0.3 mM) were prepared in anhydrous ethanol. After the dye solution was stirred for ∼1 h, it was filtered through a 0.2 μm syringe filter (with polytetrafluoroethane membrane). Prepared photoanodes were soaked in the dye solutions for ∼24 h under dark conditions to attach dye molecules to the porous-TiO_2_ surface. The dye-sensitized photoanodes were rinsed with anhydrous ethanol to remove excess dye and then dried under nitrogen.

### 2.5. DSSC fabrication

Activated platinum solution (*i.e.*, Platisol T) was spin coated at 2000 rpm for 10 s on top of the FTO substrates followed by sintering at 450 °C for 15 min to prepare the counter electrode (CE). The CE was then cooled, unassisted, for ∼40 min until it reached room temperature. The CE was assembled with the photoanode using the thermoplastic sealant. A channel of approximately 1.5 mm width (through the entire cell) was kept for electrolyte injection. The I_3_^−^/I^−^ electrolyte solution was then injected through the reserved channel in between the photoanode and CE. The channel openings were glued using a conventional hot glue gun.

### 2.6. Characterization and evaluation

Current density–voltage (*J*–*V*) characteristics of DSSCs were evaluated under an AM1.5 illumination at a light intensity of 100 mW cm^−2^. A Xenon 40 lamp (Newport 67005) with an AM1.5 filter was used as the light source. The incident photon-to-current efficiency (IPCE) was measured as a function of wavelength from 350 to 800 nm using a xenon lamp connected to a Newport monochromator. The monochromatic light was focused onto the active area of the DSSCs. The IPCE data were calibrated with a National Renewable Energy Laboratory (NREL) calibrated reference cell. UV-Vis absorption spectra were acquired using an Agilent 8453 UV-Vis spectrophotometer.

## 3. Results and discussion

### 3.1. Photovoltaic performance

Because of its nanoporous nature, an adequately sintered mesoporous TiO_2_ layer offers a large specific surface area (∼1000 times the area of projected planar surface).^17,18^ For dip-coating, the solvent carries dye molecules through the nanopores of the nc-TiO_2_ film, in order to allow covalent bonding of the dye to the TiO_2_.^19^ Because solution diffusion through nanopores is relatively slow, a long period of time (16–24 h) is necessary to fully sensitize the nc-TiO_2_*via* dip-coating. Conversely, in the vapor phase, the dye molecules can freely diffuse into the TiO_2_ nanopores to quickly produce maximum surface coverage, as described by Mallam *et al.*^6^ Moreover, to produce a conformal coating, dye molecules must reach to the end of the pores of the nc-TiO_2_ layer, for which the solvent molecules, used for dip-coating, can actually act as a barrier, preventing full dye coverage. In FCD, the gaseous dye molecules have comparatively easy access through the nanopores.

**Fig. 5:**
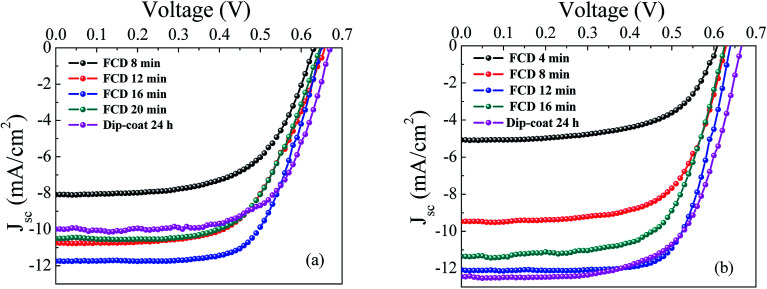
Comparison of photovoltaic performance for dip-coating and FCD-based DSSCs (a) *J*–*V* curves of L1-based DSSCs; an optimum 16 min FCD shows significantly better performance compared to 24 h dip-coating (b) *J*–*V* curves of L2-based DSSCs; an optimum 12 min FCD shows equivalent performance to 24 h dip-coating.

Fig. 5 shows the current density–voltage (*J*–*V*) characteristics curves of L1 and L2 dye-based DSSCs fabricated with dip-coating (for ∼24 h) and FCD (at different durations). Table 1 summarizes the photovoltaic performances of these DSSCs. For both L1 and L2 dyes, the short circuit current density (*J*_sc_) increases with the FCD duration up to an optimum duration. Though the molecular size of L2 is slightly larger than the L1, surprisingly, the optimum duration for L2 dye was found at a shorter deposition period (12 min) than that of L1 dye (16 min). This reveals that the specific dye structure, leading to different surface orientation, may affect the surface bonding kinetics.^4,20–22^

**Table 1:** Current density–voltage (*J*–*V*) and IPCE characteristics of L1 and L2-dye based DSSCs fabricated with FCD and dip-coating, and UV-Vis absorbance of dyes desorbed from the photoanodes

Dye		Photoanode dye loading method and duration	*J*–*V* characteristics				IPCE characteristics		UV-Vis absorbance	
			*J*_sc_ (mA cm^−2^)	*V*_oc_ (V)	FF	*η* (%)	*J*_sc_ (mA cm^−2^)	Peak IPCE (%)	Max. abs. wavelength (nm)	Absorbance (a.u.)
L1		FCD (8 min)	−8.10	0.64	0.60	3.08	−6.43	69.15	397.24	0.50
		FCD (12 min)	−10.77	0.66	0.60	4.24	−9.82	81.06	405.18	0.67
		**FCD (16 min)**	**−11.76**	**0.65**	**0.66**	**5.03**	**−11.10**	**85.51**	**412.01**	**0.85**
		FCD (20 min)	−10.55	0.65	0.61	4.22	−9.65	81.45	414.40	0.89
		Dip-coating 24 h	−10.13	0.68	0.63	4.36	−10.61	81.94	413.00	0.84
L2		FCD (4 min)	−5.10	0.61	0.60	1.86	−5.10	45.87	445.16	0.29
		FCD (8 min)	−9.52	0.63	0.65	3.87	−8.84	71.60	442.51	0.67
		**FCD (12 min)**	**−12.14**	**0.64**	**0.70**	**5.46**	**−11.80**	**88.23**	**443.16**	**0.97**
		FCD (16 min)	−11.43	0.63	0.64	4.58	−10.92	83.25	441.16	1.06
		Dip-coated 24 h	−12.53	0.67	0.64	5.35	−12.28	86.63	445.16	0.96

For prolonged FCD periods (*i.e.*, 20 min for L1, and 16 min for L2), the *J*_sc_ values decreased, leading to decreased cell efficiencies. The lower *J*_sc_ values, at longer deposition times, were likely caused by dye aggregation. Aggregation leads to non-surface bound dye molecules, resulting in photon capture without injection of the excited electron into the conduction band of TiO_2_, causing nonradiative decay of the electron from excited state to the ground state.^4,6^

Although the *J*_sc_ values increased markedly with deposition time until the optimum FCD duration was reached, the *V*_oc_s (for both L1 and L2 dyes) were little affected (with ∼0.65 V for L1, ∼0.63 V for L2). This was expected since DSSC photovoltage is determined by the difference between the conduction-band edge of TiO_2_ and the redox potential of the electrolyte,^23^ and generally not dependent on dye coverage. However, there is a small difference in *V*_oc_ between FCD and dip-coated DSSCs for both L1 and L2 dyes. In both cases, dip-coated *V*_oc_s were slightly higher than those of FCD. The origin of this difference is not fully understood. However, though the *V*_oc_s for dip-coated DSSCs were slightly higher compared to the FCD DSSCs, the PCEs (of dip-coated DSSCs) were lower, especially for L1-dye based DSSCs. The PCE for dip-coated L1 DSSC was significantly lower than that of the FCD (4.36% compared to 5.03%), mainly due to the lower *J*_sc_ (10.13 mA cm^−2^ compared to 11.76 mA cm^−2^). The low *J*_sc_ from dip-coating is likely due to non-conformal dye coating and/or inadequate sensitization. The maximum PCE and *J*_sc_ (5.2%, and 12.8 mA cm^−2^ respectively) for L1-based dip-coated DSSCs was reported by Liu *et al.*^23^ However, they used *tert*-butanol-acetonitrile (1 : 1) as a dye solvent, and 1 mM deoxycholic acid (DCA) as a co-adsorbent. In our study, we used anhydrous ethanol as a solvent and no co-adsorbent, which might limit solubility and increase aggregation, respectively, of the L1 dye. For L2-based DSSCs, the efficiency attained by dip-coating (5.35%) was similar to that of FCD (5.46%). These efficiencies are comparable with the world record efficiency (5.94%).^4^ This study suggests that the dip-coating efficiency of structurally similar dyes (*i.e.*, triphenylamine in this case) may be vastly different, while FCD (allowing free diffusion of dye vapor) is free from this problem.

### 3.2. Incident photon-to-current conversion efficiency (IPCE)

**Fig. 6:**
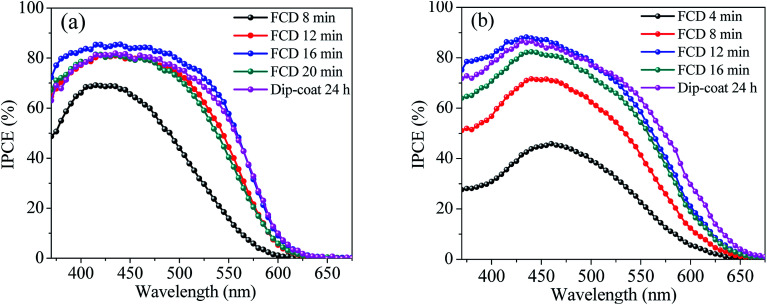
Comparison of incident photon-to-current conversion efficiency (IPCE) for dip-coating and FCD-based DSSCs (a) IPCE spectra of L1 dye based DSSCs; peak IPCE increased with increased FCD duration until reached an optimum duration of 16 min (b) IPCE spectra of L2 dye based DSSCs; peak IPCE increased with increased FCD duration until reached an optimum duration of 12 min.

To compare the incident monochromatic photon-to-current conversion efficiency (IPCE) between FCD and dip-coated DSSCs, we measured the IPCE spectra (Fig. 6) as a function of wavelength. The maximum IPCEs for L1 and L2-based DSSCs (for both FCD and dip-coating) were around 420 and 440 nm, respectively, which is consistent with Hagberg *et al.*^24^ However, there is a slight red shift in the peak IPCE for L2-based DSSC, when FCD was implemented for 4 min. The *J*_sc_ values were calculated from IPCE spectra using eqn (1)^25^ to compare with the *J*_sc_ values obtained from the *J*–*V* characteristics (Table 1).1
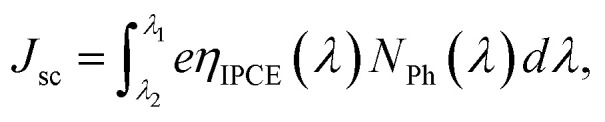
where *η*_IPCE_(*λ*) and *N*_Ph_(*λ*) are the IPCE and photon flux at wavelength, *λ*, respectively.

The calculated *J*_sc_ values, shown in Table 1, are consistent with those measured from *J*–*V* curves.

### 3.3. Dye adsorption

To study dye attachment at different durations, dye was desorbed from the photoanodes and UV-Vis absorbance of the resulting solution was measured. Table 1 shows that the absorption peak intensity increases with FCD duration, resulting from increased dye loading with increased deposition duration. It is noticeable that a similar amount of dye was desorbed from the dip-coated (∼24 h) cells and the optimized FCD (16 min for L1 and 12 min for L2) cells for both dyes. For the L1-based DSSC, though equal amount of dye was attached for 16 min FCD and ∼24 h dip-coating, there is a marked difference in the PCE, which may be due to non-conformal dip-coating sensitization. After optimum FCD duration, both the PCE and the IPCE decreased even though the amount of dye extracted increased (Table 1). This decrement is likely due to the dye aggregation, which results in decreased *J*_sc_s. This result is in agreement with the previous FCD study.^6^ There was a slight blue shift in absorbance spectra for less-concentrated L1 dye solutions (*i.e.*, solutions correspond to 8 and 12 min FCD for L1-based photoanodes). A concentration-dependent shift in absorbance spectra was also observed by Kitamura *et al.* for organic dyes.^26^ However, there is no shift in absorbance spectra for L2 dye.

### 3.4. Reduction of dye waste

Besides offering reduced deposition time, one of the major advantages of FCD is efficient use of DSSC dyes, which can be very costly. Dyes are more efficiently used for FCD in a number of ways. First, when dissolving a dye for dip-coating, even after stirring 1–2 hours, it is necessary to filter the solution to remove the undissolved coarse dye grains to ensure they don't interfere with deposition. On the contrary, FCD doesn't require dissolution or filtering of the dye, reducing effort and dye waste.

Second, dye solutions for dip-coating are typically used multiple times, with storage in between. Storing dye-solutions for longer periods (*e.g.*, >1 week) may easily lead to dye degradation (*e.g.*, oxidation). Using this solution multiple times can cause poor sensitization and results in lower PCEs. FCD eliminates this issue by using a small amount of solid dye.

Third, during dip-coating, dye solutions must be kept in the dark (as the dye molecules are very light sensitive in solution) for long durations (*i.e.*, 16–24 h). Whereas, in FCD, the vacuum chamber limits photoexcitation of the dye and the dye loading time is very small (∼20 min), so the consequences of the light sensitivity are mitigated.

Dye use was further reduced in this study compared to that of Mallam *et al.*^6^ For the Mallam *et al.* study, a relatively large amount of dye was sublimated to sensitize multiple electrodes placed at different positions within the FCD chamber, leading to less-controlled and less-reproducible sensitization. For the current study, the FCD process was modified using a single vial for each photoelectrode (Fig. 4). The metal jacket ensured the dye did not deposit on the sides of the vial within the region of the jacket. At selected temperatures and pressures, only a small amount of dye was sublimated. In addition to photoelectrode surface, a small amount of dye deposited on the sides of the vials (above the metal jacket) and on the sides of the substrate. In Mallam *et al.*^6^ study, much more dye deposited on the walls of the FCD chamber. Following FCD, vials and the substrates were rinsed sequentially with anhydrous ethanol and acetone to remove excess dye. The excess dye in the wash solution was recovered, for future use, by evaporating the ethanol and acetone to dryness.

Based on all the advantages of FCD over dip-coating, and modification of the FCD process in this study, it was estimated that approximately, 20% of the dye is necessary for FCD as compared to dip-coating.

## 4. Conclusion

Although DSSC fabrication has undergone multiple modifications, the standard dye-loading technique (*i.e.*, dip-coating) remains a major limiting issue taking more than 80% of the fabrication time. This study demonstrates that FCD with a higher vacuum (∼0.1 mTorr) can produce DSSCs with equivalent or higher PCEs compared to dip-coating for larger molecular dyes (*e.g.*, MW > 420 g mol^−1^) within a fraction of sensitization duration (∼2%) of dip-coating. The shorter FCD dye-sensitization time (*i.e.*, <20 min) was attributed to gaseous dye molecules' relatively easy diffusion capability through TiO_2_-nanopores to quickly produce maximum surface monolayer coverage. Besides the reduction of dye-loading duration, FCD demonstrated more efficient use of dye material and significant reduction of solvent use.

## Conflicts of interest

There are no conflicts to declare.

## Supplementary Material
